# Complete chloroplast genome sequences of an important horticultural orchid: *Paphiopedilum hirsutissimum* (Orchidaceae)

**DOI:** 10.1080/23802359.2019.1662752

**Published:** 2019-09-09

**Authors:** Zhe Zhao, Mingyu Li, Jian He, Jin Cheng, Lei Xie

**Affiliations:** a College of Biological Sciences and Technology, Beijing Laboratory of Urban and Rural Ecological Environment, Beijing Key Laboratory of Ornamental Plants Germplasm Innovation and Molecular Breeding, Beijing Forestry University, Beijing, China;; b School of Nature Conservation, Beijing Forestry University, Beijing, China

**Keywords:** Horticultural plant, Orchidaceae, *Paphiopedilum*, phylogenomics, whole chloroplast genome

## Abstract

*Paphiopedilum hirsutissimum* is an important ornamental orchid species with great horticultural value. The whole chloroplast genome was assembled using genome skimming data. The total length of the cp genome is 154,569 bp, comprising a large single-copy (LSC) region of 85198 bp, a small single-copy (SSC) region of 683 bp, and a pair of inverted repeat regions (IRs) of 34,344 bp each. The chloroplast genome consists of 125 functional genes, including 79 protein-coding genes, 8 rRNA genes, and 38 tRNA genes. The systematic position of the species was also inferred based on plastid phylogenomic analyses.

*Paphiopedilum* (Orchidaceae), known as the lady’s slipper orchid, is a subtropical orchid genus (Chung et al. 2006). *Paphiopedilum hirsutissimum* (Lindl. ex Hook. f.) Stein is native to crevices on shaded cliffs or rocky and well-drained places in forests or thickets in limestone areas at the elevation of 700–1500 m in northern and western Guangxi, southern and western Guizhou, southern and eastern Guizhou, northern and eastern India, Laos, Thailand, and northern Vietnam. (Liu et al. 2009; Li et al. 2015; Chen et al. 2018). It is highly variable in floral structure, which makes it an important germplasm of horticultural plant resource (Chen et al. 2018). For the purpose of understanding this species, we reported and characterized the first complete chloroplast genome of *P. hirsutissimum* using the next-generation sequencing method. In addition, we also presented a phylogenomic analysis of this species and its relatives.

We sampled the leaf material from a living individual in the greenhouse of National Engineering Research Center of Floriculture (Beijing Forestry University), which was collected from Guangxi Province (N 24.856541°, E 106.397648°, voucher no. *MY Li* 201902, BJFC). Total genomic DNA was extracted using CTAB method (Doyle and Doyle 1987). Then, second generation sequencing was performed with an Illumina HiSeq 4000 platform at Novogene (http://www.novogene.com, China). Chloroplast reads were selected out with map function of Geneious R11 (Kearse et al. 2012) using published chloroplast genomes of *Paphiopedilum* as references (Kim et al. 2015; Lin et al. 2015; Hou et al. 2018). Filtered reads were assembled using *de novo* method implemented in Geneious R11. The assembled complete chloroplast sequence was then annotated using Plann (Huang and Cronk 2015).

The chloroplast genome of *Paphiopedilum hirsutissimum* is a double-stranded circular DNA molecule with 154,569 bp in size. The cp genome has a typical quadripartite structure of a pair of inverted repeats (IRs) regions of 34,344 bp each, separated by a large single-copy (LSC) region of 85198 bp, and a small single-copy (SSC) region of 683 bp. The total GC content is 36.3% and the corresponding values of the LSC, SSC, and IR regions are 33.8%, 28.7%, and 39.4%, respectively.

The chloroplast genome consists of a total of 125 functional genes, including 79 protein-coding genes (PCGs), 8 rRNA genes, and 38 tRNA genes. Among them, 12 protein-coding (*rps*16, *atp*F, *rop*C1, *ycf*3, *rps*12, *clp*P, *pet*B, *pet*D, *rpl*16, *rpl*2, *ndh*B, *rps*12) and six tRNA genes (*trn*K-UUU, *trn*G-GCC, *trn*L-UAA, *trn*V-UAC, *trn*I-GAU, *trn*A-UGC) have introns.

Complete chloroplast genome sequences of *P. hirsutissimum* and 26 related taxa from Orchidaceae and other monocots available from GenBank were downloaded for phylogenomic analyses. Bayesian inference and maximum likelihood methods were applied for phylogenetic reconstruction (Figure 1). All the setting of sequence alignment, ML and Bayesian analyses were the same with Kim et al. (2015). In the present study, phylogenetic framework of *Paphiopedilum* as well as Orchidaceae were consistent with all the previous phylogenetic studies (Kim et al. 2015; Lin et al. 2015; Hou et al. 2018).

**Figure 1.**
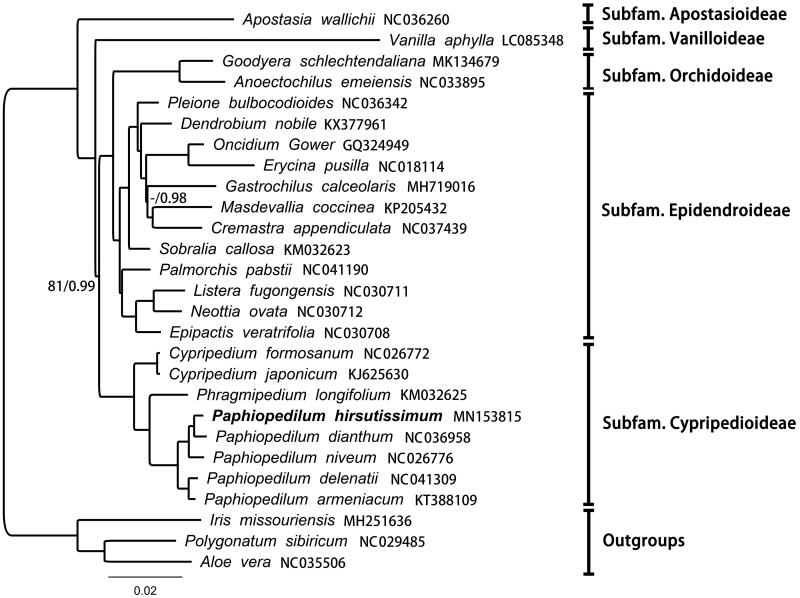
Bayesian phylogram of *Paphiopedilum hirsutissimum* and its relatives inferred from the complete chloroplast genome sequences. Branches with less than 100 ML bootstrap value and 100% PP value are shown at each node. “-” shows ML bootstrap value less than 50.
